# Physical activity and health related quality of life

**DOI:** 10.1186/1471-2458-12-624

**Published:** 2012-08-07

**Authors:** Nana Kwame Anokye, Paul Trueman, Colin Green, Toby G Pavey, Rod S Taylor

**Affiliations:** 1 Health Economics Research Group (HERG), Brunel University, Uxbridge, Middlesex, UB8 3PH, England; 2 Peninsula College of Medicine and Dentistry, University of Exeter, Veysey Building, Salmon Pool Lane, Exeter, EX2 4SG, England; 3 School of Human Movement Studies, University of Queensland, St. Lucia Campus, Brisbane, QLD 4072, Australia

**Keywords:** Health related Quality of Life, Objective measure, Self reports, Physical activity, EQ-5D

## Abstract

**Background:**

Research on the relationship between Health Related Quality of Life (HRQoL) and physical activity (PA), to date, have rarely investigated how this relationship differ across objective and subjective measures of PA. The aim of this paper is to explore the relationship between HRQoL and PA, and examine how this relationship differs across objective and subjective measures of PA, within the context of a large representative national survey from England.

**Methods:**

Using a sample of 5,537 adults (40–60 years) from a representative national survey in England (Health Survey for England 2008), Tobit regressions with upper censoring was employed to model the association between HRQoL and objective, and subjective measures of PA controlling for potential confounders. We tested the robustness of this relationship across specific types of PA. HRQoL was assessed using the summary measure of health state utility value derived from the EuroQol-5 Dimensions (EQ-5D) whilst PA was assessed via subjective measure (questionnaire) and objective measure (accelerometer- actigraph model GT1M). The actigraph was worn (at the waist) for 7 days (during waking hours) by a randomly selected sub-sample of the HSE 2008 respondents (4,507 adults – 16 plus years), with a valid day constituting 10 hours. Analysis was conducted in 2010.

**Results:**

Findings suggest that higher levels of PA are associated with better HRQoL (regression coefficient: 0.026 to 0.072). This relationship is consistent across different measures and types of PA although differences in the magnitude of HRQoL benefit associated with objective and subjective (regression coefficient: 0.047) measures of PA are noticeable, with the former measure being associated with a relatively better HRQoL (regression coefficient: 0.072).

**Conclusion:**

Higher levels of PA are associated with better HRQoL. Using an objective measure of PA compared with subjective shows a relatively better HRQoL.

## Background

Physical activity (PA) is associated with reduced risk for health conditions including coronary heart disease, cancers, diabetes, and stroke [1-3]. Despite interventions to increase PA in the English general population, the Health Survey for England (HSE) (2008) showed that only 39% of men and 29% of women in England are meeting the recommended level to be considered ‘physically active’ as defined by guidance from the Chief Medical Officer; only 6% of men and 4% of women, however, met the recommended level when PA was objectively measured [4].

Health Related Quality of Life (HRQoL), including the physical and mental well-being of an individual, is an important concept in health research and can help to inform decisions on prevention and treatment of ill health [5]. Estimates of the relationship between HRQoL and particular health states have served as inputs for economic evaluations intended to inform resource allocation decisions [6]. From a public health perspective a better understanding of how healthy lifestyles, such as uptake of PA, can influence HRQoL might help to inform policy intended to incentivise PA in the general population [5]. Evidence on the association between HRQoL and PA in the general population is limited as research, to date, has focused on specific interventions or populations with chronic conditions [7]. The dearth of evidence perhaps partly explains why economic evaluation of exercise interventions, to date, has rarely accounted for HRQoL gains directly attributable to increased exercise [8], so called process utility, and rather has focussed on the long-term effects of sustained PA on the incidence of chronic conditions.

A recent systematic review [7] showed that the few studies that have attempted to address this gap in knowledge, found a positive association between HRQoL and PA. The limited evidence base is, however, considered weak as the studies have methodological issues with respect to the measurement of PA [7,9]. The studies mainly used subjective (self-reported) measures of PA without adequate validation. Some commentators argue that regardless of appropriate validity and reliability tests, the use of subjective measures of PA are subject to overestimation [10]. The few studies [11,12] that used objective measures, however, relied on cardio-respiratory fitness, which unlike electronic devices (e.g. accelerometers) is not a direct measure of PA. As being a biological phenotype, cardio-respiratory fitness is influenced by both PA level and genetics, and the consequent interaction of both [7]. A notable exception is Hamer and Stamatakis [9] that used accelerometer data to explore the association between objectively assessed PA and subjective measures of well-being.

The present study is unique because it uses both objective and subjective measures of PA, and focuses on a broader measure of health status as it is the common endpoint in economic evaluation of public health interventions. The paper aims to explore the relationship between HRQoL and PA, and examines how this relationship differs across objective and subjective measures of PA, within the context of a large representative national survey from England. This study focuses on adults aged 40–60 years in order to: (a) relate the findings more directly to current evidence on exercise referral interventions in the UK as the literature centres on this age group; and (b) reflect the broader objectives of the programme of research exploring those interventions [8]. This research is part of a larger programme of research that explored the effectiveness and cost effectiveness of exercise referral schemes in the United Kingdom [8].

## Methods

### Data

Data came from the HSE, a routine cross sectional survey that draws a nationally representative sample of persons residing in private households in England, which is openly available. The sample and focus of the survey vary each year. Data from the 2008 survey was used in this study and included a sample of 9,191 households with 15,102 adults aged 16 or over, and a total child sample of 7,521. Households were sampled proportionately across the 9 Government Office regions of England. Sampling was based on a multi-stage stratified random sampling design that used the postcode address file as a sampling frame. To improve power of analysis, boost samples and sampling weights were employed appropriately [4]. The primary focus of HSE 2008 was PA and fitness. The method of data collection involved face-to-face interviews, self-completion, clinical and physical measurements (including objective measurements of PA via accelerometers). Tools for data collection were validated in a pilot study conducted in 2007 prior to the main survey (further details can be found in Craig et al. [4]). To compensate for seasonal variation in responses, the time period for interviews covered January-December 2008. This study draws on 5,537 observations that constitute 40–60 year olds among the adult sample.

### HRQoL

HRQoL is measured in the HSE using the summary measure of health state utility value derived from the validated EuroQol-5 Dimensions (EQ-5D) [13]. These utility scores were generated using the descriptive system of the EQ-5D questionnaire (UK version), a standard HRQoL instrument with preference weights which are attached to combinations of responses. The EQ-5D descriptive system describes HRQoL in five dimensions (i.e. mobility, self-care, usual activities, pain/discomfort, and anxiety/depression) with each dimension including three levels: no problems, some/moderate problems, and severe/extreme problems. Different health states are created from the responses to the descriptive system of the EQ-5D by combining one level from each of the dimensions. A tariff is then applied to these health states to generate utility scores [14]. The utility scores usually range from ‘1’ (perfect health) to ‘0’ (death), with states perceived to be worse than death having a negative utility score.

### Physical activity

PA was accessed via a composite indicator, reflecting a combination of types of PA (i.e. walking, housework, occupational activity, and sports and exercise) and captured through subjective, and objective measurements. In HSE 2008, the subjective measure of PA was assessed through a validated questionnaire and the objective measure the most widely used accelerometer, actigraph (model GT1M), which is relatively portable (due to its small size) and associated with low running costs [4]. Accelerometer is a favoured method of objective measurement of physical activity given that it has an increased capacity to capture varied movements [4]. A pilot study was conducted to examine the feasibility of using the actigraph was and it was found to perform well. In the main survey, the actigraph was worn (at the waist) by a randomly selected sub-sample of the HSE 2008 respondents (4,507 adults – 16 plus years). Respondents were told to wear the actigraph during waking hours for 7 consecutive days following an initial discussion with interviewers who explained the use of the device and gave respondents phone numbers to call for assistance when faced with difficulties using the device. A book was given to participants to log daily use of the actigraph (duration of use/non-use). Daily use was considered ‘valid’ if the actigraph was worn for at least 10 hours. Kine software (3.0.98) was used to analyse the raw accelerometry data to generate a standardised measures. Further details on the use of accelerometer in the HSE 2008 can be found in Craig et al. [4].

To test the robustness of the relationship between PA and HRQoL across specific types of PA, separate measures were included for specific types of PA (i.e. walking, and sports and exercise). Data for walking included self-reports on all walking (regardless of intensity) ranging from country walks, walking to and from work/school, and any other walks. Sports and exercise covered self-reports on activities such as swimming, cycling, aerobics, workout at a gym, running, team sports, and press/sit-ups. These two types of PA were chosen and analysed separately because they reflect the main types of exercise referral interventions [8].

Each PA measure was operationalised as a binary variable that takes the value of one if ‘physically active’ (minimum of 90 minutes of at least moderate intensive PA was undertaken per week) or zero otherwise (not physically active). This definition of being ‘physically active’ is consistent with the literature on exercise referral interventions and PA for health guidance [15]. Based on previous research [11,12,16], we hypothesise that being ‘physically active’ would be positively correlated with HRQoL.

### Covariates

The covariates were socio-demographic, economic, health and other variables that in previous research had been shown to correlate with HRQoL: gender, age, income, educational qualification, employment status, ethnicity, marital status, house tenure, smoking status, drinking status, region of residence, urbanisation, general health status, BMI, morbidities (e.g. problems with heart, muscoskeletal, ear, vision, mental, hypertension, stroke, diabetes), psycho-social wellbeing (measured via General Health Questionnaire scores) [17-23].

### Analysis

Means (standard deviation - SD) and proportions were calculated for continuous and categorical data respectively. Chi square and Fischer’s exact tests were used to check the association between HRQoL (dependent variable) and dummy variables representing item non-response for independent variables in order to examine the mechanisms under which the missing data occurred (i.e. missing completely at random or not) [24]. If the pattern of missing data did not occur completely at random (i.e. HRQoL is significantly associated with item non-response for independent variable(s), a regression-based imputation method was used to replace missing values of continuous variables and a dummy variable specifying item-non response added. For the categorical variables, item non-response was included in the omitted category and a dummy variable for item non-response created [25].

To account for censoring in the measurement of HRQoL [26], Tobit regressions with upper censoring at 1.0 and robust standard errors were employed to model the relationship between HRQoL and PA controlling for covariates. Separate models were fitted for each indicator of PA to avoid unstable estimates resulting from the collinearity among those indicators. Hence four different models were estimated: model 1 (walking); model 2 (sport and exercise); model 3 (objective measurement); model 4 (subjective measurement). Hereafter, the models will be referred to by these names. Each model had two versions: (a) model excluding missing observations; and (b) model including imputed missing observations. To allow comparability between subjective and objective measures of PA, models 3 and 4 were estimated using the same sample (i.e. those with data for both subjective and objective measures).

The models were estimated with sampling weights that were calculated as the inverse of the probability of being a respondent in a household multiplied by the household weight which accounts for non-responding households [4]. Specification errors and goodness of fit of models were examined using the linktest [27], and Akaike Information Criteria (AIC) and Bayesian Information Criteria (BIC) [28] respectively. Pseudo R^2^ was computed by calculating the R^2^ between the predicted and observed values [29] and multicollinearity among independent variables was assessed [30,31]. Threshold for statistical significance was set at ≤ 0.10 and analysis was undertaken in 2010 using Stata version 10.

## Results

### Description of sample

Table 1 shows that the mean health state utility score (HQRoL) was 0.86 (Standard Deviation: 0.23; upper limit:1; lower limit:-0.484) and few individuals had limiting illness (23%). The proportion of ‘physically active’ individuals ranged from 12% (via objective measurement) to 45% (via subjective measurement). Based on self-reports, 46% of respondents whose physical activity were objectively measured (n = 873) were physically active compared with 44% for those who did not provide such data (n = 4652). Majority of the sample were White (91%), female (55%), married and living with partners (66%), educated (81%), and employed (76%). Few people were obese (26%) or smokers (22%) though majority (85%) were ‘drinkers of alcohol’. Average annual household income was £35,591 (SD: 29,210).

**Table 1:** Descriptive statistics of variables (adjusted for missing observations)

**Variables**	**Observations**	**Mean(SD) /%**
Dependent variable (HRQoL)		
EQ-5D	5453	0.86 (0.23)
Missing	84	1.5
Independent variables (PA)		
Walking		
*Active*	873	15.8
*Inactive*	4664	84.2
Sports and exercise		
*Active*	660	11.9
*Inactive*	4877	88.1
Objective measurement*		
*Active*	102	11.5
*Inactive*	783	88.5
*Missing*	4652	84
Subjective measurement**		
*Active*	2452	44.4
*Inactive*	3067	55.6
*Missing*	18	0.3
Independent variables (covariates)		
Age	5537	50(6.2)
Gender		
*Male*	2519	45.5
*Female*	3018	54.5
Marital status		
*Other*	30	0.5
*Married (living with partner)*	3618	65.3
*Single*	735	13.3
*Separated*	208	3.8
*Divorced*	816	14.7
*Widowed*	135	2.4
Income	4535	35591.2 (29210)
*Missing*	1002	18.1
*Income (missing observations imputed for)*	5537	35008.3 (26987.7)
Drink alcohol		
*Yes*	4702	84.9
*No*	823	14.9
*Missing*	12	0.2
Smokers		
*Yes*	1206	21.8
*No*	1926	34.8
*Missing*	2405	43.4
BMI		
*Underweight (under 18.5)*	30	0.5
*Normal (18.5 – 25)*	1487	26.9
*Overweight (25 – 30)*	1885	34
*Obese (30+)*	1418	25.6
*Missing*	717	13
Ethnicity		
*White*	5029	90.8
*Mixed*	44	0.8
*Asian*	260	4.7
*Black*	140	2.5
*Chinese*	28	0.5
*Other*	17	0.3
*Missing*	19	0.3
Education		
*Nvq4/nvq5/degree or equivalent*	1228	22.2
*Higher education below degree*	746	13.5
*Nvq3/GCE ‘A’ level equivalent*	749	13.5
*Nvq2/GCE ‘O’ level equivalent*	1404	25.4
*Nvq1/CSE other grade equivalent*	239	4.5
*Foreign/other*	53	1.0
*No qualification*	1102	19.9
*Missing*	16	0.3
Employment status		
*Employed*	4215	76.1
*Unemployed*	163	2.9
*Retired*	259	4.7
*Other economically inactive*	884	16
*Missing*	16	0.3

*The distribution of objective measurement (missing observations imputed for) is: active (111;2%) inactive(5426;98%) ** The distribution of subjective measurement (missing observations imputed for) is: active (2465; 44.5%) inactive (3072; 55.5%). If restricted to observations with values for objective measurement, the distribution of subjective measurement (missing observations imputed for) is: active (408; 46.1%) inactive (477; 53.9%).

### Missing observations

HQRoL had 84 missing observations (2%), and these were dropped creating an effective sample of 5,453 observations. All the independent variables except walking; sports and exercise; age; marital status; region of residence and urbanisation had missing observations. Most variables had around 1% of missing data. The objective measure of PA had more missing observations (84%) because as per HSE procedures only a random subset of the sample was selected for the objective measurement although the subjective measurement was based on the entire adult sample.

The HQRoL for individuals, who had missing values for social class; BMI; and smoking status, were significantly different from those who did not. However, HQRoL for individuals with missing values for the indicators of PA were not different from those who did not. Hence findings on the association between PA and HRQoL is not expected to be significantly affected by the inclusion (or not) of missing observations.

### Regression models

Table 2 shows the reduced regression models estimating the correlation between PA and HRQoL, controlling for covariates. Results are presented for models (excluding missing observations) because they provide better fit and specification, and were consistent with those of models that included missing observations.

**Table 2:** Estimation results of regression models (reduced models without missing observations)

**Independent variables**	**Dependent variable (Health Related Quality of Life)**							
	**Model 1**^**a**^	**SE**^**d**^	**Model 2**^**a**^	**SE**^**d**^	**Model 3**^**a**^	**SE**^**d**^	**Model 4**^**a**^	**SE**^**d**^
	**Coefficient (95% CI**^**b**^**)**^**c**^		**Coefficient (95% CI**^**b**^**)**^**c**^		**Coefficient (95% CI**^**b**^**)**^**c**^		**Coefficient (95% CI**^**b**^**)**^**c**^	
Walking								
*Active*	0.026 (−0.003, 0.055)*	0.014						
*Inactive (reference)*								
Sports and exercise								
*Active*			0.034 (−0.000, 0.068)**	0.018				
*Inactive (reference)*								
Objective measure								
*Active*					0.072 (−0.014, 0.157)*	0.044		
*Inactive (reference)*								
Subjective measure								
*Active*							0.047 (0.022,0.072)***	0.013
*Inactive (reference)*								
Constant	1.456 (1.286, 1.626)***	0.090	1.159 (0.980,1.338)***	0.091	1.252 (1.017, 1.487)***	0.120	1.393 (1.277,1.510)***	0.059
Number of observations	3957		3957		873		874	

^a^ All models [Model 1 (physical activity indicator: walking), Model 2 (physical activity indicator: sports and exercise), Model 3 (physical activity indicator: objective measure of physical activity), Model 4 (physical activity indicator: subjective measure physical activity] are adjusted to gender, age, income, educational qualification, employment status, ethnicity, marital status, house tenure, smoking status, drinking status, region of residence, urbanisation, general health status, BMI, morbidities (e.g. problems with heart, muscoskeletal, ear, vision, mental, hypertension, stroke, diabetes), psycho-social wellbeing (measured via General Health Questionnaire scores) .^b^95% Confidence Interval; ^*c*^Significance level of 1%(***), 5%(**) , 10%(*); ^d^robust standard error.

Higher levels of PA are associated with better HRQoL. Compared with inactive individuals, being ‘physically active’ via objective or subjective measure was associated with a higher HRQoL but the objective measure was associated with 53% extra HRQoL gain (coefficient(*r*):0.072) relative to the latter (coefficient(*r*):0.047).

The positive association between HQRoL and PA was consistent across specific types of PA as individuals who were ‘physically active’ via sports and exercise (coefficient(*r*)::0.034) or walking (coefficient(*r*):0.026) had better HRQoL than inactive individuals.

### Model diagnostics

The specification error tests show that the models had good specification and that additional statistically significant regressors could only be found by chance (Table 3). The models’ estimates could be considered stable as no sign of multicollinearity was found, with average variance inflation factors and tolerance levels at 1.2 and 0.8 respectively. A reasonable proportion (between 10% and 40%) of variation in HRQoL was explained by the models as indicated by the pseudo R^2^ . Model 3 appears to have the best fit according to Akaike Information Criteria (AIC) and Bayesian Information Criteria (BIC) values.

**Table 3:** Model diagnostics

**Models**	**Multicollinearity tests**		**Specification test**	**Pseudo R**^**2**^	**AIC**	**BIC**
	***VIF***	***Tolerance***	***P>|t|***			
Model 1	1.05 - 1.45	0.68 – 0.97	0.063	0.429	3247.5	3360.6
Model 2	1.04 - 1.55	0.65 – 0.95	0.084	0.430	2697.7	2798.3
Model 3	1.00 - 1.22	0.82 – 0.99	0.845	0.092	841.7	889.5
Model 4	1.06 - 1.23	0.81 – 0.94	0.205	0.446	1936.9	2050.1

## Discussion

The results support the hypothesis that higher levels of PA are associated with better HRQoL. This relationship is consistent across different measures and types of PA. Participation in walking and sports and exercise are correlated with a modest effect on HRQoL. However, differences in the magnitude of HRQoL benefit associated with objective and subjective measures of PA are noticeable, with the former measure being associated with a relatively better HRQoL. An explanation for this could be that people tend to over-report their participation levels in PA [32], for example, 90 minutes of at least moderate intensive PA captured via a subjective measure might actually equate to a shorter duration or a less intensive period of exercise when measured objectively. The potential impact of this is that it yields a downward bias in econometric estimation with respect to subjective measures of PA, meaning that the link between HRQoL and PA is underestimated. This is further supported by descriptive statistics that indicate that people considered physically active via objective measure had slightly better mean HRQoL value (0.918) than individuals considered physically active via self-reports (0.916).

A key question is whether in order to achieve more accurate estimations, objective rather than subjective measures ought to be favoured. We are inclined towards a cautious approach, allowing for further corroboration of the findings because of a number of reasons. First, there was discrepancy in the reference periods of data collection for the two measures, subjective data was based on four weeks prior to survey date while objective data was based on a seven day period after the survey date. Second, the objective measure used herein, accelerometry, do not capture all types of PA with equivalent precision tending to underestimate activities like cycling and walking up stairs [4].

It is important that the limitations of the analysis are acknowledged. First, as a cross sectional study, the findings point to an association between HRQoL and PA and that no conclusions on causality can be drawn. Therefore, we cannot conclude whether participation in PA leads to higher HRQoL or improved HRQoL leads to a lifestyle (e.g. absence of limiting illnesses) more enabling for participation in PA. Nonetheless, the consistency of our findings across different types of PA strengthens the need for further research into the causal pathways between PA and HRQoL using longitudinal datasets. Second, the findings may not be generalisable to other age groups given the emphasis on adults aged 40–60 years. However, the findings are consistent with previous evidence on the relationship between PA and HRQoL [11,12,16]. Further confidence can be drawn from the findings because all regression models had good specification and fit.

The findings suggest that there is a relationship between PA and HRQoL. However, the nature of the study means that it is not possible to accurately dissect the nature of this relationship. It is important to consider the constituents of the HRQoL gains associated with PA captured in this study. Participation in PA generally tends to be associated with two main types of benefits i.e. physical benefits (reduced risk for ill-health conditions, improved fitness levels) and psychological benefits (e.g. mental simulation during participation, improved psychological health) [33,34]. Given the cross sectional nature of our study and the fact that ill-health conditions were controlled for in the analysis, the benefits captured may more likely be psychological benefits. However, we are not able to determine the exact nature of these benefits. For example, the improvements in HRQoL associated with PA might be considered to be ‘process benefits’ that arise from engagement in PA. These might occur due to the process of participating (increased social interactions resulting from group participation or time spent outdoors), improved self-esteem (e.g. positive perceptions of competences and the physical-self) or biologic mechanisms (increased endorphin levels as a result of PA). Further research is needed to understand the relationship between PA and HRQoL and this may need to comprise both quantitative and qualitative methods.

Related to this, is the degree to which these benefits are lasting which also warrants further exploration as its crucial to how HRQoL gains are converted into outcomes (e.g. quality adjusted life years or ‘QALY’s’) in an economic evaluation. Sustaining PA levels is necessary if the risks of long-term conditions are to be modified. However, it remains unclear whether any process benefits associated with PA, as defined above, are sustained over time or whether these are associated with moving from a sedentary lifestyle to an active lifestyle, meaning individuals accrue a ‘one-off’ benefit to HRQoL. One might consider that HRQoL improvements arising from improved self-esteem are transitory whereas improvements resulting from biologic mechanisms might be sustained over time.

Notwithstanding these limitations, the findings here do indicate the potential for generating policy relevant information on promotion and evaluation of PA programmes. Programmes aimed at improving the uptake of PA might encourage participation rates by making people aware of the potential gains (which might occur sooner than later) associated with participation. In terms of economic evaluation, the findings here reinforce the need for investigation into the impact of the inclusion of such HRQoL gains. Previous analyses have suggested that including such benefits, alongside the longer-term benefits resulting from reduced incidence of long-term conditions, will lead to an improvement in the cost effectiveness of interventions designed to increase PA [35]. However, further research is required to understand the relationship between HRQoL improvements which might result directly from participation in PA as differentiated from improvements that result from sustained participation and reduced incidence of long-term conditions. Only by examining these relationships will we be in a position to accurately determine the cost effectiveness of interventions designed to promote PA.

## Conclusion

Higher levels of PA are associated with better HRQoL. Using an objective measure of PA compared with subjective shows a relatively better HRQoL. Given the measurement errors associated with subjective measures, a key question is whether in order to achieve more accurate estimations of HRQoL, objective rather than subjective measures of PA ought to be favoured. We are inclined towards a cautious approach, allowing for further corroboration of the findings particularly because of discrepancy in the reference periods of data collection for the two PA measures.

## Competing interests

All authors declare that they have no competing interests.

## Authors’ contribution

Nana Kwame Anokye and Paul Trueman undertook the analysis with support from Colin Green, drafted the first manuscript and coordinated its revision. Toby Pavey and Rod Taylor provided an advisory role and assisted in drafting and revising the manuscript. All authors read and approved the final manuscript.

## Pre-publication history

The pre-publication history for this paper can be accessed here:

http://www.biomedcentral.com/1471-2458/12/624/prepub
